# Fulminant Histoplasmosis Presenting as Pyrexia of Unknown Origin in Immunocompetent Adult Diabetic Patient

**DOI:** 10.5005/jp-journals-10071-23154

**Published:** 2019-04

**Authors:** Prakash Shastri, Pallav Gupta

**Affiliations:** 1 Critical Care and Emergency Medicine, Sir Gangaram Hospital, New Delhi, India; 2 Department of Histopathology, Sir Gangaram Hospital, New Delhi, India

**Keywords:** Bone marrow, Histoplasmosis, Immunocompetent

## Abstract

Opportunistic infections are commonly seen in immunocompromised individuals such as those suffering from HIV infection, organ transplant recipients and connective tissue disorders. Histoplasmosis is one of the fungal opportunistic infections which is usually seen in immunosuppressed individuals but can also be seen in immunocompetent patients with frequent involvement of adrenal glands. We present a case of adult diabetic patient presenting as pyrexia of unknown origin in which bone marrow aspiration and biopsy lead to diagnosis of histoplasmosis without adrenal involvement.

**How to cite this article:** Shastri P, Gupta P. Fulminant Histoplasmosis Presenting as Pyrexia of Unknown Origin in Immunocompetent Adult Diabetic Patient. Indian J Crit Care Med 2019;23(4):193–195.

## INTRODUCTION

Histoplasmosis is caused by dimorphic fungi histoplasma capsulatum and is considered an opportunistic infection seen in immune compromised individuals. It resides in soil contaminated with bat and birds excreta and the infection is acquired by inhalation. It can manifest clinically as pulmonary, primary cutaneous or disseminated disease.^1^ Although rare in Indian subcontinent, sporadic cases have been reported from eastern and south India.^2^ We report a case of histoplasmosis in non-HIV Immunocompetent male presenting as pyrexia of unknown origin.

## CASE REPORT

A 63-year-old male, a known hypertensive and diabetic for 20 years presented with high-grade fever associated with chills. Fever was persistent since last three months and was associated with weakness and weight loss (10 kg). It was not associated with cough, cold, loose motions, vomiting, pain abdomen or headache. He was investigated for infectious causes, however no specific source was found. Despite treatment with antibiotics his symptoms persisted. He was then investigated for connective tissue disorders which revealed high ACE level for which corticosteroids were given in the previous hospitalization (not ours). However fever did not subside. Contrast enhanced computed tomography (CECT) of thorax showed solitary pulmonary nodule with centrilobular emphysema while ultrasound abdomen was unremarkable. Serum protein electrophoresis was normal, fibroscan was 20.9. A PET scan was also done which showed sellar mass of 2.5 × 2.2 × 1.6 cm with sellar expansion. Hormonal work up was normal. Based on CECT findings and positive quantiferon Gold essay, he was started on anti tubercular therapy (ATT) outside our hospital. He did not show response to the treatment and ATT was stopped after one month.

On presentation to our hospital patient was found to be drowsy, oriented to time, place and person, blood pressure was 108/74 mm hg, pulse rate 92/min, SPO2 88%. On examination, pallor was present, chest examination revealed bilateral basal crepitations with normal air entry. CVS examination was normal. Abdomen was soft, non tender without any obvious organomegaly. He was admitted and started on IV antibiotics, IV hydrocortisone (replacement doses) and supportive medications. A provisional differential diagnosis of sarcoidosis, granulomatous polyangitis and infective etiology was kept. Routine investigations were normal (Table 1). Scrub typhus IgM, Weil Felix test, *Brucella* antibody and viral markers (HIV, HBsAg and HCV) were negative. Urine routine was within normal limits. Urine and blood cultures were sterile (Table 1). HbA1c was 6%. ACE level was elevated (221 U/L). Serum calcium levels were high which were managed with saline diuresis and calcitonin injection. MRI pituitary was done to rule out granulomatous hypophysitis. MRI was suggestive of pituitary macroadenoma. Vasculitis markers including C-ANCA, P-ANCA were negative. Patient condition however deteriorated rapidly and he developed breathing difficulty with fall in blood pressure after which he was shifted to ICU and ventilated. In view of unexplained fever a bone marrow aspiration and biopsy were planned.

**Table 1:** Investigations at admission

Hemoglobin	8 g/dL
Total leukocyte count	5100/mm^3^
Platelets	1 lakhs/mm^3^
HbA1c	6%
ACE	221 U/L
HbsAg, HCV HIV	Negative
Urine and blood culture	Sterile

**Fig. 1:**
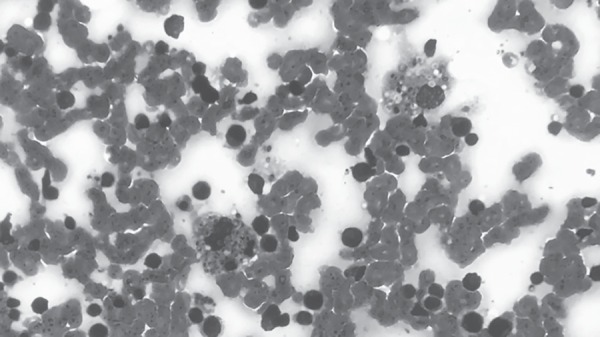
Intracellular budding yeast forms within histiocytes in bone marrow biopsy (Hematoxylin and Eosin, 1000x original magnification)

**Fig. 2:**
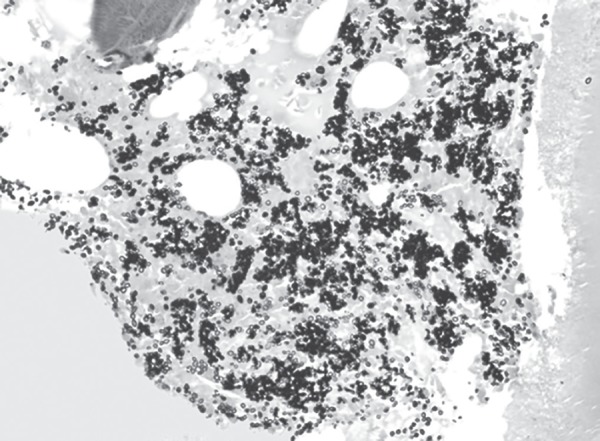
Gomori silver stain highlighting budding yeast forms of histoplasma (GMS, 1000x original magnification)

**Figs 3A and B:**
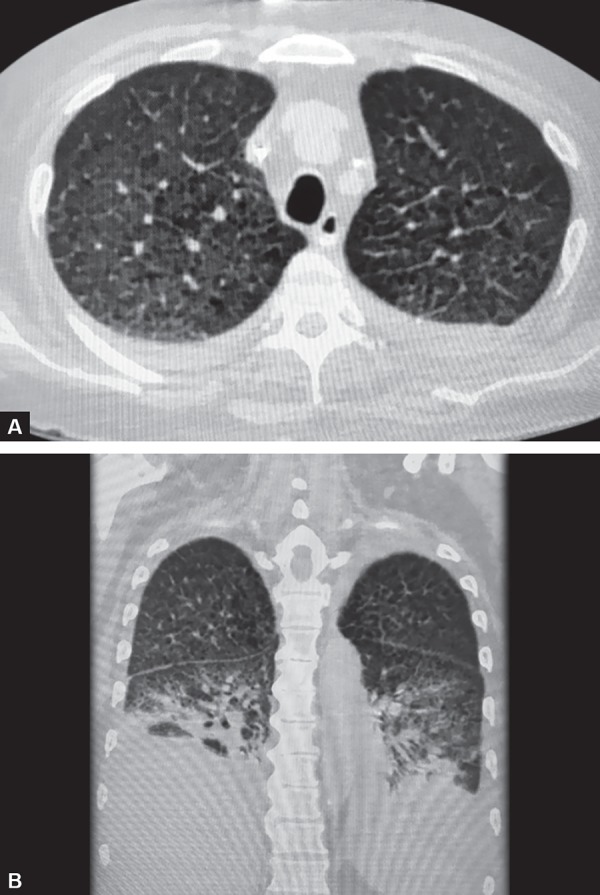
HRCT thorax showing bilateral pleural effusion with extensive emphysematous changes

Bone marrow aspirate revealed presence of intracellular budding yeast forms within histiocytes and also extracellularly. They were 2–4 µm in diameter and had pseudocapsule (Fig. 1). Gomori silver methanamine stain highlighted these organisms (Fig. 2). Morphological findings were diagnostic of histoplasmosis.

Chest X-ray showed bilateral heterogenous haziness with reticulo-nodular pattern. For further evaluation, HRCT thorax was done, which was suggestive of bilateral pleural effusion, extensive emphysematous changes with few mediastinal lymph nodes largest being 1.5 cm in diameter (Fig. 3). CD4 and CD8 T cell counts were within normal limits.

With a clinical diagnosis of disseminated histopalasmosis patient was started on IV amphotericin B. Aggressive management was continued. On 10th day of admission patient went into asystole and expired.

## DISCUSSION

Histoplasma capsulatum is a dimorphic fungus, i.e., it exists in two forms, the infective mycelial or mould form in the soil and the yeast form in human macrophages.^3^ It is inhaled as microconidia/ mycelial fragments into lungs where it is converted to yeast form. Yeast forms are usually intracellular but rarely can be seen in the extracellular space. The clinical manifestations vary from asymptomatic, acute or chronic pulmonary histoplasmosis and disseminated histoplasmosis (spread to other organs and bone marrow). The progressive disseminated histoplasmosis usually affects the immunocompromised patients particularly those affected with HIV. It indicates impaired cell mediated immunity.^8,9^ The extent of disease depends on the host immune response. In immunocompromised individuals disseminated histoplasmosis can be seen to involve the reticuloendothelial system including liver, spleen, bone marrow apart from involving the adrenal glands.^4^ Cutaneous lesions, pancytopenia and anemia are more common with histoplasmosis in immunocompromised patients. Diagnosis is usually established by bone marrow aspiration/biopsy in immune compromised patients and by adrenal gland biopsy or culture studies in Immunocompetent patients.^5^

Histoplasmosis has a worldwide distribution but endemic in central America particularly the Ohio and Mississippi areas.^6^ Only a handful of cases of histoplasmosis in immunocompetent patients have been reported from India, majority of them being in diabetic patients. In India first case of histoplasmosis was reported from Calcutta in year 1954 by Panja and Sen.^7^ Exposure to birds, working in moist areas and mining have been observed risk factors for developing histoplasmosis.^6^ It has been reported that histoplasmosis in India manifests usually as fever, weight loss and mucocutaneous lesions.^8^ In immunocompetent Indian patients, adrenal was most frequently involved followed by mucocutaneous lesions, spleen, lymphodes and lungs. Bone marrow involvement is infrequently seen. Our patient was diabetic with no other comorbidities. He was an urban dweller and had no history of exposure to birds and was native of nonendemic zone. He had involvement of liver and lungs on CT thorax and abdomen and the diagnosis was confirmed on bone marrow aspiration and biopsy.

Histoplasmosis in immunocompetent individual is usually asymptomatic in 95-99% cases and sometimes manifest as minor illness such as cough, fever and chest pain. Chest radiograph shows hilar/mediastinal lymphadenopathy. These changes may mimic tuberculosis and may mislead the clinician.^5,10^

The definitive diagnosis can be done by microscopic examination of tissues or by culture. Other modalities are detection of antibodies and antigens. Immunodiffusion test to detect precipitins diagnostic antigens, the H and M antigen, is most widely available techniques for diagnosis, this assay has specificity of 70–100%. Complement fixation tests for histoplasmosis have a sensitivity range of 70–90%, but are less specific than immunodiffusion (70–80%).^10^

We present an unusual case of histoplasmosis presenting as pyrexia of unknown origin in a non HIV Immunocompetent male patient. Unlike the previous reported cases in immunocompetent patients there was no adrenal enlargement and presentation was in the form of pyrexia of unknown origin. The present case highlights that histoplasmosis should be considered in the differential diagnosis in patients with pyrexia of unknown origin especially those who have not responded to ATT. In the absence of any organomegaly bone marrow examination can lead to early diagnosis and treatment which can be life saving in such patients.
